# SonoGames: sounds of the right kind introducing gamification into radiology training

**DOI:** 10.1186/s13104-021-05761-y

**Published:** 2021-08-30

**Authors:** Maria Fatima Ali, Naila Nadeem, Farah Khalid, Naveed Muhammad Anwar, Ghulam Nabie, Charles Docherty

**Affiliations:** 1 Centre for Innovation in Medical Education, Aga Khan University Hospital, National Stadium Road, Karachi, 74800 Sindh Pakistan; 2 Department of Radiology, Aga Khan University Hospital, Karachi, Pakistan; 3 Department of Medicine, Aga Khan University Hospital, Karachi, Pakistan

**Keywords:** Education, Games, Radiology, Self-efficacy, Simulation, Ultrasound

## Abstract

**Background:**

Radiology as compared to other fields of medicine has lagged, in incorporating modern training modalities such as gamification and simulation into its teaching curriculum.

**Objective:**

This study aims to evaluate effectiveness of simulation-based teaching in collaboration with gamification. Bandura’s conception of self-efficacy was used to provide qualitative assessment of participants’ learning process through training event. Modified competitive game-based teaching methodology was utilized in an experimental study conducted for radiology residents. Workshop was divided into two sessions, first being three interactive didactic lectures followed by three competitive rounds. All participants were required to fill pre and post-self-efficacy questionnaire along with an activity evaluation form.

**Results:**

Significant self-efficacy scores were calculated for simulation-based stations of knowledge assessment and hands-on stations. Whereas significant association was also found between gender and knowledge assessment in communication skill (0.054), Professionalism (0.004), and general knowledge (0.018). Similarly, noteworthy correlation was found between gender and all hands-on skills. In conclusion, study reported an overall increase in knowledge of post-test scores compared to pre-test scores due to use of gamification in combination with simulation-based teaching which shows a positive role in clinical training. However, further consideration is needed to improve process of integrating simulation in clinical training of participants.

**Supplementary Information:**

The online version contains supplementary material available at 10.1186/s13104-021-05761-y.

*"By sticking it out through tough times, people emerge from adversity with a stronger sense of efficacy."*

–Albert Bandura

*Encyclopedia of Human Behavior, 1994*

## Introduction

Medical education is dissemination of knowledge to healthcare professionals regarding real world scenarios that they might face in their respective fields [1]. Practical training brings with itself some dilemmas. One such conundrum is safety and wellbeing of patients, while providing optimal care. Other side of the coin requires repeated exposure to better understand and respond to clinical situations [2]. Another factors is the necessity of doctors to be well versed with teamwork and good communication skills piled on to basic need of knowledge and skill [3, 4].

It is vital that medical education cannot and should not lag compared to other fields of learning, thus incorporation of simulation-based training (SBT) in clinical learning is compulsory. Moreover, simulation is a technique to help either replace and/or augment learning experience that is gained from real situations. SBT is immersive in characteristics, aimed to draw participant into a task or setting as they were experiencing it in an actual setting [5, 6].

Clinical SBT is an ideal solution to problem posed in medical education regarding patient safety versus leering and exposure of doctor, with ability to diminish risk associated with patient while providing a life-like scenario. Techniques used in SBT are used for training purposes and evaluation of competencies [7, 8]. It may seem novel, however, SBT has been majorly used in aviation and military, whereas in medicine it has been used in anesthesiology [2, 5, 6]. Impact of simulation on how medicine is taught has already led to changes in curriculums for healthcare providers, where participants have opportunity to practice, develop and master skills, via a process of try and repeat [9–11]. SBT also allows one to refresh their skills or to practice unique and uncommon clinical presentations and be prepared for when they arise without putting patient at risk. This depiction of conditions from textbooks adds a layer of intrigue to scenario while developing heightened levels of enthusiasm. There are many educationists and pioneers who believe that SBT increases efficiency skill and knowledge [12–15].

Use of simulation as an advent of teaching and training in radiology has been a relevant factor dating as long back as case conference which is a part of radiology training. This method introduced two distinct types of simulation which were visual or auditory. Images are displayed to participants; they review and assess images then work towards a differential diagnosis and treatment. It is identical to what radiologist would experience in a routine day, thus adding high fidelity to exercise. With evolution of technology, mannequins were used as simulators to augment training process [15–18].

In medicine and radiology where sifting through images and reports can numb individual, resulting in a lack of concentration, disconnection with knowledge that is being disseminated. Hence, it was identified that a non-conventional method of teaching (gamification) had potential to be effective for students and residents [19]. Many institutes also implemented a game-based (GB) educational system, which was enthusiastically received by participants, showing increased levels of understanding of ultrasound in clinical practice while also increasing their capabilities [20].

Main obstacle in simulation-based education (SBE) comes with evaluation of its outcomes, along with problem of assessing effectiveness. Hence, Bandura proposed method of assessing self-efficacy (SE) [21]. World of education has also seen adrift from using routine teaching methods to more hands-on and interactive teaching modalities with incorporation of entertaining way to learn, such as competitions being held and conversion of lecture room into a game room, having students both enjoy and become more engaged in their learning [22].

Centre for Innovation in Medical Education (CIME) at The Aga Khan University Hospital (AKUH) has proposed to incorporate teaching modality of gamification in a fun and interactive way, by holding first ever Sonogames (SG) in Pakistan, where radiology residents test their knowledge against each other while making whole processes enjoyable.

### Study implication and objective

In Pakistan, GB simulation training is not widely available and prevalent. This study provided a motive for Healthcare institutions to work on improving the understanding and integrating SBT programs in all specialties of health science. Objective of this study is to assess Radiology residents’ knowledge, hands-on skills, and integration of knowledge into clinical decision making. Furthermore, it aims to evaluate SE of participants as a measure for competency using GB simulation training program.

## Main text

### Methodology

#### Study design, population and setting

An experimental study was conducted to assess perception, technical skills, knowledge, and SE of participants of SG. Target population was College of Physicians and Surgeons Pakistan (CPSP) registered radiology residents from four hospitals of Karachi. SG was conducted at CIME, AKU. Exemption was taken from institutional ethics review committee.

#### Sampling method and sample size

Non-Probability purposive sampling was used with sample size of 30 residents who participated in SG by assuming 50% prevalence rate of SE with 95% confidence interval.

##### Inclusion criteria

- Radiology residents registered with CPSP, who had yet to pass any part of their FCPS Part II examination.
- Participants who registered for workshop.

##### Exclusion criteria

- Participants who didn’t attend lecture, all three rounds including briefing, simulation, and debriefing sessions.

#### Self-efficacy and potential implications

SE is the belief we have in our abilities, to meet challenges and complete a task successfully. [21]. Tool used to evaluate SE is a pre-and post-training questionnaire using Scale of 0–100 [23]. Both questionnaires had similar questions and response options. Teaching design allowed participants to be put through rigorous sessions of knowledge recall in pressure situations and time-sensitive environments.

#### Data collection and analysis

Written consent was obtained from all 30 participants. They were instructed to fill out pre-training questionnaire assessing their expertise and knowledge before practicing. Questionnaire was validated by faculty of radiology, which also obtained psychometric evaluation on their discretion. Groups were then subsequently debriefed about their performances.

After completing the event, participants were asked to fill the post SEQ. This helped them to reflect on knowledge they had gained so that they could compare their SE before and after session by filling in post-training questionnaire portion along with an activity evaluation form.

Data was entered in SPSS (Statistical Package for Social Sciences) version 19.0. Frequency and percentages were reported for quantitative variables, whereas qualitative variables were reported as statements. Independent and paired *T*-Test were used to find statistical significance in pre and post self-efficacy scores.

#### Planning and preparation

CIME in collaboration with Department of radiology arranged SG. Majority of information was collected from ‘SonoGames: effect of an innovative competitive game on education, perception, and use of point‐of‐care ultrasound’ [20] and ‘SonoGames: an innovative approach to emergency medicine resident ultrasound education’ [24].

A team of five Radiologists from department of radiology at AKUH were selected to act as organizer, coordinator, moderator, and judges. Team developed SG by dividing into three interactive lectures following three rounds conducted over four hours. All competition questions and simulation scenarios were written and reviewed by team. Organizing team of radiologists were ably supported by technical team of CIME for smooth working of simulators. Whereas, media and marketing team promoted event.

#### Competition structure

There were three rounds carried out on same day to remove chance of bias for teams getting more time to study up and have an unfair advantage. Teams were challenged in timed trials made up of unique and innovative GB rounds to test their skills and knowledge.

At the end, a grand debriefing and feedback session of all participants was conducted. Winning team was awarded medals whereas all participants were given 4.00 Accreditation Council for Continuing Medical Education (AACME) credit hours’ certificate.

### Results

#### Demographic details

Thirty residents took part in this workshop, out of which 17 were female and 13 were male. Eight participants were from 1st and 2nd year residency program. 22 participants were from 2nd and 3rd year residency program.

##### SE score in relation to knowledge assessment and hands-on station

Significant association was found among all SE questions which highlights that SBT along with gamification has a positive influence on participants SE. Pre and post scores in medical knowledge showed significant change with p-value of < 0.001. Scores of reading an ultrasound images, and making a provisional diagnosis were also significant with a p-value of < 0.001 for both. However, difference in pre and post scores for reading an X-ray (13) and making a provisional diagnosis (13) was less than that of scores in medical knowledge (24).

Second part of questionnaire included questions on SE in relation to activities performed during hands-on stations. A significant association of p-value < 0.001 was found in all variables of self-efficacy. Highest difference in SE score was found in performance of hip ultrasound on a neonate (34) compared to the score seen in making a final diagnosis which had least difference (16). Details can be found in Table 1.

**Table 1:** Self-efficacy score in relation to knowledge and hands-on assessment

	Pre	Post	Difference	p-value
**Medical knowledge variables**				
	48.6	72.6	24.0	< 0.001
Practice based learning and improvement	50.0	72.6	22.6	
Interpersonal and communication skills	60.1	74.9	14.8	
Professionalism	66.6	82.0	15.4	< 0.001
Follow principles of ethics and confidentiality in interacting with patients and health care team				
**General knowledge**				
Explain/read an X-ray	62.0	75.0	13.0	< 0.001
Explain/read an ultrasound	61.0	80.0	19.0	
Make a provisional diagnosis from the findings in radiograph	62.0	75.0	13.0	
**Hands-on variables**				
**Fast chase station**				
Perform a transvaginal ultrasound	49.6	72.6	23.0	< 0.001
Perform a hip ultrasound on a neonate	36.6	70.6	34.0	
Follow the proper protocol	54.0	72.3	18.0	
Identify the findings	48.3	70.0	21.7	
Define the findings	53.3	72.0	18.7	
Make a proper diagnosis	54.0	71.3	17.3	
**Blind partner station**				
Perform an obstetric ultrasound	59.6	76.0	16.4	
Perform a post-delivery ultrasound	59.3	75.6	16.3	
Explain/decipher the ultrasound findings	59.3	76.6	17.3	
Make a provisional diagnosis	58.3	74.3	16.0	
Make a final diagnosis	55.6	72.0	16.4	
Use proper ultrasound terminologies	60.0	76.6	16.6	
**Communication station**				
Identify different sorts of presentation of pregnancy	56.3	76.0	19.7	
Convey the reason for your missed diagnosis	3.3	71.0	17.7	

#### SE score of knowledge assessment and hands-on skills in relation to gender

Considering variation in genders with regards to response of SE pre and post questionnaire, parameter of medical knowledge between males and females showed SE mean difference scores of 26.1 and 22.4 respectively. While second parameter measured in questionnaire of practice-based learning and improvement gave mean values of 26.9 for males and 19.41 for females. Third variable titled interpersonal and communication skills, gave a mean of 20.7 in males and 12.35 in females with a significance p-value of 0.054 respectively. Furthermore, with regards to professionalism where mean values were 20.8 in men and 7.65 in women with a significance p-value of 0.004. Last section was of general knowledge, where mean scores were 20.13 for men and 10.88 for women with a significance p-value of 0.018.

In Fast chase skills station, SE mean difference of male participants was reported to be 27.18 and 18.33 of females with a significance of 0.024. Blind partner skill station reported male score as 23.91 and female score as 10.83 with 0.001 of significance. In communication skills, self-efficacy mean difference of males was 26.54 and 12.65 of female participants with a significance of 0.001. Details are mentioned in Table 2.

**Table 2:** Self-efficacy score of knowledge assessment and hands-on skills in relation to gender

	Male					Female					p-value
	Pre		Post		Diff	Pre		Post	Diff		
**Knowledge assessment variables**											
Medical knowledge	44.62		70.70		26.10	51.76		74.12	22.40		0.372
Practice based learning and improvement	44.62		71.54		26.9	54.12		73.53	19.41		0.072
Interpersonal and communication skills	54.62		75.38		20.70	66.47		78.82	12.35		**0.054**
Professionalism	41.54		62.31		20.80	47.06		54.71	07.65		**0.004**
General knowledge	52.82		72.95		20.13	68.82		79.71	10.88		**0.018**
**Hands-on skills variables**											
Fast chase	38.21	65.38		27.18		57.84	76.18			18.33	**0.024**
Blind partner	47.18	71.09		23.91		67.55	78.38			10.83	**0.001**
Communication	43.46	70**.**00		26.54		63.53	76.18			12.65	**0.001**

##### Activity assessment of feedback

**Fig. 1:**
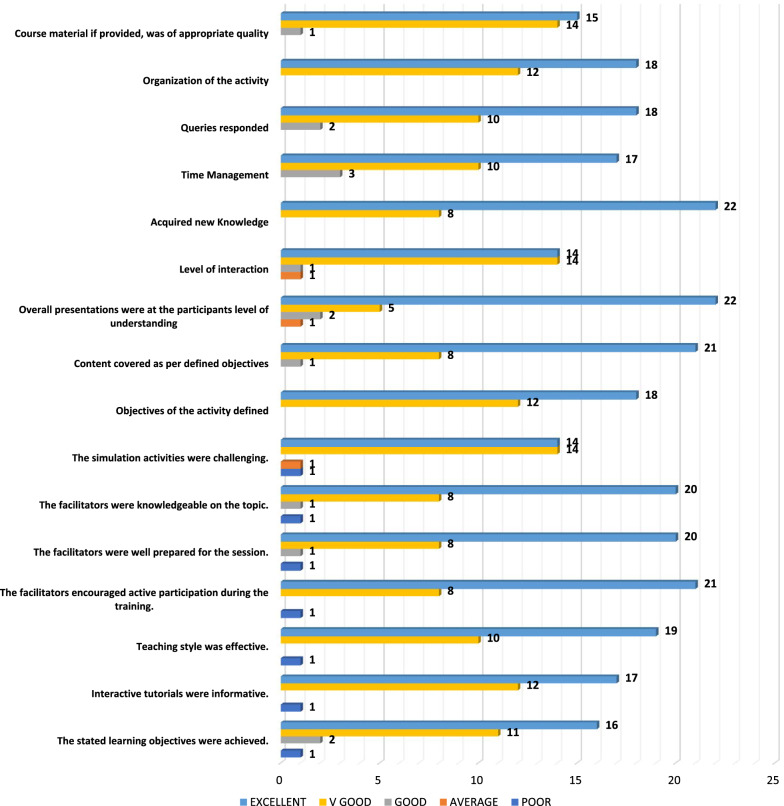
Activity assessment of feedback

Seventeen of participants stated that interactive tutorials were informative whereas 14 participants said that simulation activities were very challenging. Further details are reported in Fig. 1

All participants reported that program met their expectations and sessions were applicable to their job also that they would recommend this program to others. Further details are reported in Additional file 1.

### Discussion

Our results were noteworthy as we found that participation in SG had a positive effect on perception and understandings of residents across knowledge and clinical skills. 73% of participants of our study reported that SG helped them to acquire new knowledge while similar (80% of participants) was quoted by a study that recruited residents of Emergence Medicine (EM) [20]. Pilot study conducted on EM interns also reported similar results to ours with 81% of the participants stating an improvement in ultrasound knowledge [25].

GB simulation activities conducted in SG were rated as excellent (53%) and very good [40%] of participants. In EM residents study, 90% of participants said that hands-on games were an effective educational modality [20]. Study conducted at Stanford University states that activities like SG are beneficial as a training platform for those who have just started their residency [25].

Our study also helped residents to master art of communication. Significant association between communication, professionalism, and SE scores of all participants was reported. SG contributed in improving communication skills of EM interns of pilot study. They further stated that EM needs efficient communication skills and this approach of teaching helped them progressing through training [25]. Study conducted in Boston registers similar findings where radiology residents and fellows reported an increase in post communication mean score. Similar study also stated that participants gave a good score to quality of lecture whereas 56.6% of residents in our study said that quality of tutorials was excellent [17].

In our study, post mean score of knowledge assessment is higher than pre mean score in all participants similar to Chen et al. where an increase in post test scores by an average of 10 points was reported [26].

Positive feedback was given by all participants. “Event was good, and I thoroughly enjoyed this approach of learning” said one female resident. A participant who worked for a private hospital said “This idea is novel for us as we do not have access of learning through simulation. This course has helped me in increasing my ultrasound skills”.

In conclusion, study reported an overall increase in knowledge of post-test scores compared to pre-test scores. Use of gamification in combination with SBT shows a positive role in clinical training. However, this field needs further consideration to better the process of integrating simulation in clinical training of participants.

## Limitations

- Confined to data of one specialty.
- Not all participants were familiar with SBT and simulators.
- Number of participants was low.
- Results cannot be generalized for targeted population.
- Measurements of changes in the variables were obtained soon after event.

## Supplementary Information

**Additional file 1.** Additional Information such as preparation of Sono Games, Self-Efficacy Questionnaire used for the activity and data for analysis including charts and figures are reported in additional file.

## Data Availability

The datasets used and/or analysed during the current study available from the corresponding author on reasonable request.

## Abbreviations

SBT: Simulation based training
SBE: Simulation based education
SE: Self-efficacy
SG: Sono–games
CIME: Centre for innovation in medical education
AKUH: Aga Khan University Hospital
EM: Emergency medicine
PMDC: Pakistan medical and dental council
CPSP: College of Physicians and Surgeons Pakistan
CHK/DUHS: Civil Hospital Karachi/DOW University of Health and Science
SUIT: Sindh institute of urology and transplantation
LNH: Liaquat national hospital
AACME: Accreditation council for continuing medical education
