# Supplementary material for: SonoGames: sounds of the right kind introducing gamification into radiology training
Source: BMC Res Notes. 2021 Aug 30;14:341. doi: 10.1186/s13104-021-05761-y (PMC8404289; doi:10.1186/s13104-021-05761-y)
Supplement: Supplementary file 1 — Additional file 1. Additional Information such as preparation of Sono Games, Self-Efficacy Questionnaire used for the activity and data for analysis including charts and figures are reported in additional file. [file 13104_2021_5761_MOESM1_ESM.docx]

# Additional file 1

# Additional Information regarding Sono-Games

**Teams**

Five competing teams of six residents each from four hospitals of Karachi were formed. Residents were divided into teams according to their level of experience and knowledge. Each team nominated one member as team leader and was allotted a name. The leader assigned each member for different tasks and everyone participated equally.

**Interactive tutorials**

Session started off with three Interactive lectures which were delivered by three different faculties, having command knowledge on their designated topics.

- Gynecological ultrasound – Female imaging
- Obstetrical ultrasound – Female imaging
- Neonatal hip ultrasound – Pediatrics

**Round 1- Quiz**

Participants were shown different scenarios and ultrasound images followed by multiple-choice questions with four possible options. They were given a minute to respond to each question. Scores were tallied and disclosed at the end of the round. Questions were created from following categories.

***General knowledge (25%)****:* Basic ultrasound principles and clinical indications.

***Diagnostic (30%)****:* Diagnosis based on images and clinical scenarios.

***Management (45%)****:* Treatment plan based on view of clinical scenario and ultrasound image.

Round 2 **– Hands-on sessions**

Teams were rotated through five stations of four different simulation-based scenarios and one communication station. Faculty were assigned to respective stations for moderating scenario and ranking teams based on their performances. Each station had 10 points. A standardized 24-minute format was used at each station: 3 minutes for briefing and rules, 12 minutes for task completion, 5 minutes for feedback and debriefing and a 4-minute interval for rotation.

During debriefing period following an experiential learning activity, faculty reflected, reviewed, and discussed task with goal of improving individual and team clinical skills and judgment.

Five stations of Round 2 are briefly described below:

***Blind Partner I & II.***

There were two stations of Blind partner, each with a different case. Hands on scanning was done with SonoSimulator. Representative participant was provided with a list of “taboo” words not to be used, following which they performed ultrasound of given cases, described images, and explained findings verbally to other team members. Rest of members could not ask any questions and were facing opposite side, listening to findings, and had to come up with diagnosis; write it down on answer sheet provided.

***Scoring criteria.***

Correct diagnosis received ten points, whereas negative marking for use of a taboo word and cross communication.

***Fast Chase I & II.***

There were two stations of Fast Chase, one on Hip Sonography in newborns & Infants and other one on General Pathology Transvaginal Ultrasound Training model. Each member of the team was tested for hands-on skills on MINDRAY Ultrasound machine by scanning images of the given cases on simulators.

***Scoring criteria.***

Meet and greet 2 points

Technical ability and descriptive of findings 6 points

Diagnosis 2 points

***Radiologist: The Best Communicators.***

One station was setup to assess communication skills of participants. A member of each time was assigned for station. Participants were made to act out given case scenario on communicating with primary clinician for missed diagnosis and how to break bad news to patient. This tested ethical and moral values where participants were asked to explain reason for going through with a wrong diagnosis. All team members were required to give feedback on how to improve communication skills.

***Scoring criteria*.**

Builds relationship, Opens the discussion, explain reason in truthful manner, discuss possible reason for missing.

Two assessment tools:

A binary checklist

0 = Not done, 1 = done

Global rating scale

1= Unsatisfactory, 2= Acceptable, 3= Fair, 4= Good, 5= Very Good, 6= Excellent, 7=Outstanding

Round 3 **– Rapid fire round / Literature quiz**

Prior to commencement of SG, all participants were provided reading material, selected based on their quality, methodology along with relevance of conclusions. Each member of 5 teams had 2 minutes to answer as many ultrasound-related questions as possible from given pre reading material. Total points were recorded and displayed at the end of event.

# APENDIX I: TABLES

**TABLE 01: DEMOGRAPHIC DETAILS**

| **VARIABLES** | **FREQUENCY** | **PERCENTAGE** |
| --- | --- | --- |
| **Gender**  Male  Female | 13  17 | 43.3  56.7 |
| **Residency Year**  I  II  III  IV | 8  8  7  7 | 26.6  26.6  23.3  23.3 |
| **Institute**  AKUH  CHK/DUHS  SIUT  LNH | 9  9  8  4 | 30.0  30.0  26.6  13.3 |

**TABLE 02: ACTIVITY ASSESSMENT OF FEEDBACK**

| S.NO | VARIABLES | 1Poor | 2Average | 3Good | 4Very Good | 5Excellent |
| --- | --- | --- | --- | --- | --- | --- |
|  |  |  |  |  |  |  |
|  | The stated learning objectives were achieved. | 1 (3.30%) | 0 | 2 (6.60%) | 11 (36.6%) | 16 (53.3%) |
|  | Interactive tutorials were informative. | 1 (3.30%) | 0 | 0 | 12 (40%) | 17 (56.6%) |
|  | Teaching style was effective. | 1 (3.30%) | 0 | 0 | 10 (33.3%) | 19 (63.3%) |
|  | The facilitators encouraged active participation during the training. | 1 (3.30%) | 0 | 0 | 8 (26.6%) | 21 (70%) |
|  | The facilitators were well prepared for the session. | 1 (3.30%) | 0 | 1 (3.30%) | 8 (26.6%) | 20 (66.6%) |
|  | The facilitators were knowledgeable on the topic. | 1 (3.30%) | 0 | 1 (3.30%) | 8 (26.6%) | 20 (66.6%) |
|  | The simulation activities were challenging. | 1 (3.30%) | 1 (3.3%) | 0 | 12 (40%) | 16 (53.3%) |
|  | Objectives of the activity defined | 0 | 0 | 0 | 12 (40%) | 18 (60%) |
|  | Content covered as per defined objectives | 0 | 0 | 1 (3.30%) | 8 (26.6%) | 21 (70%) |
|  | Overall presentations were at the participants level of understanding | 0 | 1 (3.3%) | 2 (6.60%) | 5 (16.6%) | 22 (73.3%) |
|  | Level of interaction | 0 | 1 (3.3%) | 1 (3.30%) | 14 (46.6%) | 14 (46.6%) |
|  | Acquired new Knowledge | 0 | 0 | 0 | 8 (26.6%) | 22 (73.3%) |
|  | Time Management | 0 | 0 | 6 (20%) | 10 (33%) | 17 (56.6%) |
|  | Queries responded | 0 | 0 | 2 (6.6%) | 10 (33%) | 18 (60%) |
|  | Organization of the activity | 0 | 0 | 0 | 12 (40%) | 18 (60%) |
|  | Course material if provided, was of appropriate quality | 0 | 0 | 1 (3.3%) | 14 (46.6%) | 15 (50%) |

## TABLE 03: EVENT FEEDBACK

| **S.NO** | **VARIABLES** | **YES** | **NO** | **NOT SURE** |
| --- | --- | --- | --- | --- |
|  | Did the program meet your expectation | 30 | - | - |
|  | Were the sessions applicable to your job | 30 | - | - |
|  | Will you recommend this program to others? | 30 | - | - |

## Equipment & simulators

| **SIMULATOR/ EQUIPMENT** | **MANUFACTURER** | **MODEL #** |
| --- | --- | --- |
| SonoSim Live Scan (24) | SonoSim Ultrasound training Solution | -- |
| Nursing Mannequins (25) | 3B Scientific | Model P10 |
| Infant hip sonography training phantom (26) | Kyoto Kagaku | Model US-13 41914-000 |
| General Pathology Transvaginal Ultrasound Training model (27) | CAE Blue Phantom | Model BPOB1220 |
| Ultrasound Machine (28) | Mindray | Model TE 7 |

# APENDIX II: CHARTS

## CHART 01: BAR CHART – PRE AND POST SCORE OF KNOWLEDGE ASSESSMENT IN RELATION TO GENDER

**CHART 02: SELF-EFFICACY SCORE OF HANDS-ON SKILLS IN RELATION TO GENDER**

## CHART 03: EVENT FEEDBACK

**CENTRE FOR INNOVATION IN MEDICAL EDUCATION**

**THE AGA KHAN UNIVERSITY**

**SONOGAMES: Sounds of the right kind**

**SELF-EFFICACY QUESTIONNAIRE**

**CONSENT:** The purpose of this research is to evaluate the improvement in self-efficacy of new interns and residents after completing the boot camp. Filling out this form will indicate that you have given your consent to participate in this research voluntarily.

**Profession: ________________________ Year of Residency: _____________________**

**Department: _______________________ Name of Institute: ________________________**

**Gender: ___________________________**

**THIS IS NOT A TEST AND THE ANSWERS WILL BE KEPT CONFIDENTIAL. THEREFORE, PLEASE ANSWER THE QUESTIONS BELOW HONESTLY.**

**SELF-EFFICACY RESPONSE SCALE**

______________________________________________________________________________

0 10 20 30 40 50 60 70 80 90 100

I cannot do moderately certain highly certain

at all I can do I can do

**NOTE:** USE THE ABOVE SCALE FROM 0 TO 100 TO RATE *HOW CERTAINLY* YOU COULD/CAN PERFORM THE FOLLOWING OBJECTIVES BEFORE AND AFTER SONOGAMES.

| **BEFORE** |  | **AFTER** |
| --- | --- | --- |
| **MEDICAL KNOWLEDGE** | | |
| *How certain are you that you could:* | | |
|  | use up to date evidence and apply appropriate state of the art diagnostic techniques to meet the imaging needs of patients, referring physicians, and the health care system. |  |
| **PRACTICE BASED LEARNING AND IMPROVEMENT** | | |
| *How certain are you that you could:* | | |
|  | Investigate and evaluate your own patient care, appraisal and assimilation of scientific evidence and improvements in patient care. |  |
| **INTERPERSONAL AND COMMUNICATION SKILLS** | | |
| *How certain are you that you could:* | | |
|  | Effectively communicate with patients, colleagues, referring physicians and other members of the health care team concerning imaging appropriateness, informed consent, safety issues, and results of imaging tests or procedures. |  |
| **PROFESSIONALISM** | | |
| *How certain are you that you could:* | | |
|  | Follow principles of ethics and confidentiality and consider religious, ethnic, gender, educational, and other differences in interacting with patients and other members of the health care team. |  |
|  | Place patient’s interests above one’s own |  |
| **GENERAL KNOWLEDGE** | | |
| *How certain are you that you could:* | | |
|  | Explain/ read an X-ray. |  |
|  | Explain/ read an ultrasound. |  |
|  | Make a provisional diagnosis from the findings in radiograph. |  |
| **FAST CHASE STATION** | | |
| *How certain are you that you could:* | | |
|  | Perform a transvaginal ultrasound. |  |
|  | Perform a hip ultrasound on a neonate. |  |
|  | Follow the proper protocol. |  |
|  | Identify the findings. |  |
|  | Define the findings. |  |
|  | Make a proper diagnosis. |  |
| **BLIND PARTNER STATION** | | |
| *How certain are you that you could:* | | |
|  | Perform an obstetric ultrasound. |  |
|  | Perform a post-delivery ultrasound. |  |
|  | Explain/decipher the ultrasound findings. |  |
|  | Make a provisional diagnosis. |  |
|  | Make a final diagnosis. |  |
|  | Use proper ultrasound terminologies. |  |
| **COMMUNICATION STATION** | | |
| *How certain are you that you could:* | | |
|  | Identify different sorts of presentation of pregnancy. |  |
|  | Convey the reason for your missed diagnosis. |  |
